# European multinational outbreak of *Salmonella* Umbilo linked to rocket salad and baby spinach traced to buffalo farms in Italy, 2024 to 2025

**DOI:** 10.1186/s13567-025-01663-0

**Published:** 2025-12-18

**Authors:** Rossana Ferraro, Sara Petrin, Alfonso Gallo, Giovanni Cenere, Silvano Salaris, Domenico Alfano, Carmelo Morena, Giovanna Serluca, Anna Balestrieri, Yolande Therese Rose Proroga, Antonio Guarnieri, Sabato De Vita, Maurizio Della Rotonda, Donato De Nicola, Lisa Barco, Giorgio Galiero

**Affiliations:** 1 National Reference Centre for Hygiene and Technologies of Water Buffalo Farming and Productions, Istituto Zooprofilattico Sperimentale del Mezzogiorno, Portici, Italy; 2 Italian National Reference Laboratory for Salmonella, Istituto Zooprofilattico Sperimentale Delle Venezie, Padua, Italy; 3 Animal Welfare and the Fight Against Maltreatment (CRiBBAM), Regional Reference Centre for Biosecurity, Caserta, Italy; 4 Executive Task Force Prevention and Veterinary Public Health, Region Campania, Naples, Italy; 5 Farm Animal Veterinarian, Salerno, Italy

**Keywords:** Rocket salad, baby spinach, water buffalo, *Salmonella* Umbilo, biosecurity, genomic sequencing

## Abstract

Following RASFF Alert 2024.7033 (*Salmonella* Umbilo in rocket from Italy), an investigation was conducted on three buffalo farms in Salerno, Southern Italy, located near the horticultural company implicated in the production of contaminated rocket salad and baby spinach linked to the outbreak. *Salmonella* Umbilo was isolated from faecal samples of buffalo calves in one of the three farms, and whole-genome sequencing confirmed a genetic match with the outbreak strain. Irrigation channels positioned close to animal housing were identified as potential contamination pathways. Corrective measures were promptly implemented to minimise the risk of further contamination.

## Introduction, methods, and results

Between July and September 2024, a multinational food-borne outbreak was reported in several European countries, including Germany, Austria and Denmark. The outbreak, which involved approximately 200 confirmed human cases, was attributed to *Salmonella enterica* serotype Umbilo (antigenic formula 28:z10:e,n,x hereafter *S*. Umbilo) [1]. Epidemiological and microbiological investigations established a link between the cases and the consumption of contaminated rocket salad and baby spinach, with traceback analysis identifying a single horticultural facility in Italy as the source of contamination [1]. The implicated company is located in the province of Salerno, Campania region (Southern Italy) known for producing fresh-cut, ready-to-eat vegetables [2]. This area is also characterised by a high density of buffalo farms, which supply milk for mozzarella cheese production [3]. In buffalo farms, *Salmonella* spp. represents a significant and widespread health concern, causing severe economic losses [4, 5]. Buffalo calves are the category most frequently affected by *Salmonella* spp., which generally causes gastroenteritis in these animals, with mortality rates reaching up to 70% within the first month of life [6, 7]. *Salmonella* spp. infections in buffalo are caused by diverse serovars, with no evidence of species specificity [8]. According to data collected by the Regional *Salmonella* Typing Centre (Ce.Ti.Sa) of the Istituto Zooprofilattico del Mezzogiorno, the most frequently isolated *Salmonella* spp. serotypes from buffalo farms in the Campania region over the past 5 years (2020–2024) were *S*. Agona (*n* = 15), *S*. Give (*n* = 12), *S*. Goldcoast (*n* = 8), *S*. Typhimurium (*n* = 7) and *S*. Livingstone (*n* = 7). In addition, during the same period other less commonly detected serotypes were *S.* Enteritidis (*n* = 4) and *S.* Umbilo (*n* = 2) (personal communication).

This study aims to elucidate the contamination pathways of rocket salad and baby spinach in the *S*. Umbilo outbreak described in the RASFF notification ‘2024.7033 *S*. Umbilo in rocket from Italy’ [1].

To identify potential sources of *Salmonella* spp. contamination in salad crops, an epidemiological investigation was conducted on three buffalo farms located upstream of the cultivated fields, where buffaloes are permanently kept in paddocks without access to external areas. A targeted sampling plan was developed and implemented in each farm, involving the collection of various matrices to enable the isolation and characterisation of any *Salmonella* spp. strains present, in accordance with the indications provided by Regulation (EU) 179/2025 [9].

The investigation was conducted between December 2024 and February 2025 by the Italian competent veterinary authorities as a part of official controls. The implemented sampling plan was designed in accordance with the Protocol for the management of a Salmonellosis Outbreak in Dairy Cattle Farms defined by National Reference Laboratory for *Salmonella* (Istituto Zooprofilattico Sperimentale delle Venezie) [10]. The sampling procedures were established on the basis of on-site inspection findings and the number of animals present on each farm.

Diagnostic investigations specifically focused on individual animals at higher risk of *Salmonella* spp. shedding, namely neonatal calves (1–28 days old) and buffalo cows in the peripartum period or immediately post-partum. The number of calves faecal samples collected reflected the herd size and was determined according to an expected prevalence of > 10%.

From the three farms, pooled faecal samples were collected from these animals, along with bulk milk, complete milk replacer feed and animal manure. Moreover, a single drinking water sample was collected from farm 3, resulting in a total of 111 collected samples.

All samples from different matrices were sent to the laboratory (Istituto Zooprofilattico Sperimentale del Mezzogiorno) for the isolation of *Salmonella* spp. and subsequent strain typing. Faecal and animal manure samples were tested using the UNI EN ISO 6579-1:2020 method [11]. Bulk milk samples and complete milk replacer feed samples were analysed using the AFNOR BRD 07/06–07/04 method [12]. Lastly, the drinking water sample was analysed according to the UNI EN ISO 19250:2013 method [13].

Serotype identification was performed according to the Kauffmann–White–Le Minor scheme, and genomic sequencing was conducted for further characterisation on a selection of *Salmonella* spp. isolates.

Genomic DNA from isolates grown on tryptose agar was extracted using a commercial column-based kit (QIAamp DNA Mini, QIAGEN). Libraries for whole genome sequencing were prepared using the Nextera XT DNA sample preparation kit (Illumina) following the manufacturer’s instructions. High-throughput sequencing was performed on a MiSeq instrument, resulting in 300 bp long paired-end reads. Sequences were analysed according to the guidelines for reporting whole genome sequencing-based typing data through the EFSA One Health WGS System [14]. A core genome multi locus sequence typing (cgMLST) scheme approach (INNUENDO scheme, 3225 loci [14]) was followed to assess genetic relatedness among the investigated isolates and the strains isolated from a clinical case and a baby spinach sample, as described in [1].

For the genomic analyses in this context, sequences of isolates from animal faeces were considered as belonging to a cluster, if they showed up to five alleles (allelic difference, AD) from the reference sequences 2024-FWD-00070 described in [1]. A microbiological assessment of irrigation channel water was conducted at farm 3 according to UNI EN ISO 9308-2:2014 – Water quality – Enumeration of *Escherichia coli* and coliform bacteria – Part 2: Most Probable Number (MPN) method. The IDEXX Colilert® system with Quanti-Tray/2000 was employed, based on the MPN principle, to determine the concentration of *E. coli*.

*Salmonella* spp. was isolated from 25 faecal samples, all collected from buffalo calves, with no clinical signs observed at the time of sampling. All other sample types – including faecal samples from adult buffaloes, bulk milk, complete milk replacer feed, animal manure and drinking water – tested negative. These results are detailed in Table 1.

**Table 1:** **Distribution of positive and negative samples for *Salmonella*** **spp. in tested matrices, Italy, 2024 to 2025 (*n*** = **111 samples)**

Samples	Negative	Positive	Total
**Buffalo calf faeces**	49	25	74
Farm 1	3	4	7
Farm 2	27	10	37
Farm 3	19	11	30
**Adult buffalo faeces**	20	0	20
Farm 1	5	0	5
Farm 2	8	0	8
Farm 3	7	0	7
Animal manure^*^	4	0	4
Farm 1	1	0	1
Farm 2	1	0	1
Farm 3	2	0	2
**Bulk milk**	9	0	9
Farm 1	3	0	3
Farm 2	4	0	4
Farm 3	2	0	2
**Complete milk replacer feed**	3	0	3
Farm 1	1	0	1
Farm 2	1	0	1
Farm 3	1	0	1
**Drinking water**	1	0	1
Farm 3	1	0	1
Total	86	25	111

*Legend* *Animal manure refers to the combined faces, urine, and bedding material from all animals, retrieved from a liquid manure tank

**Figure 1:**
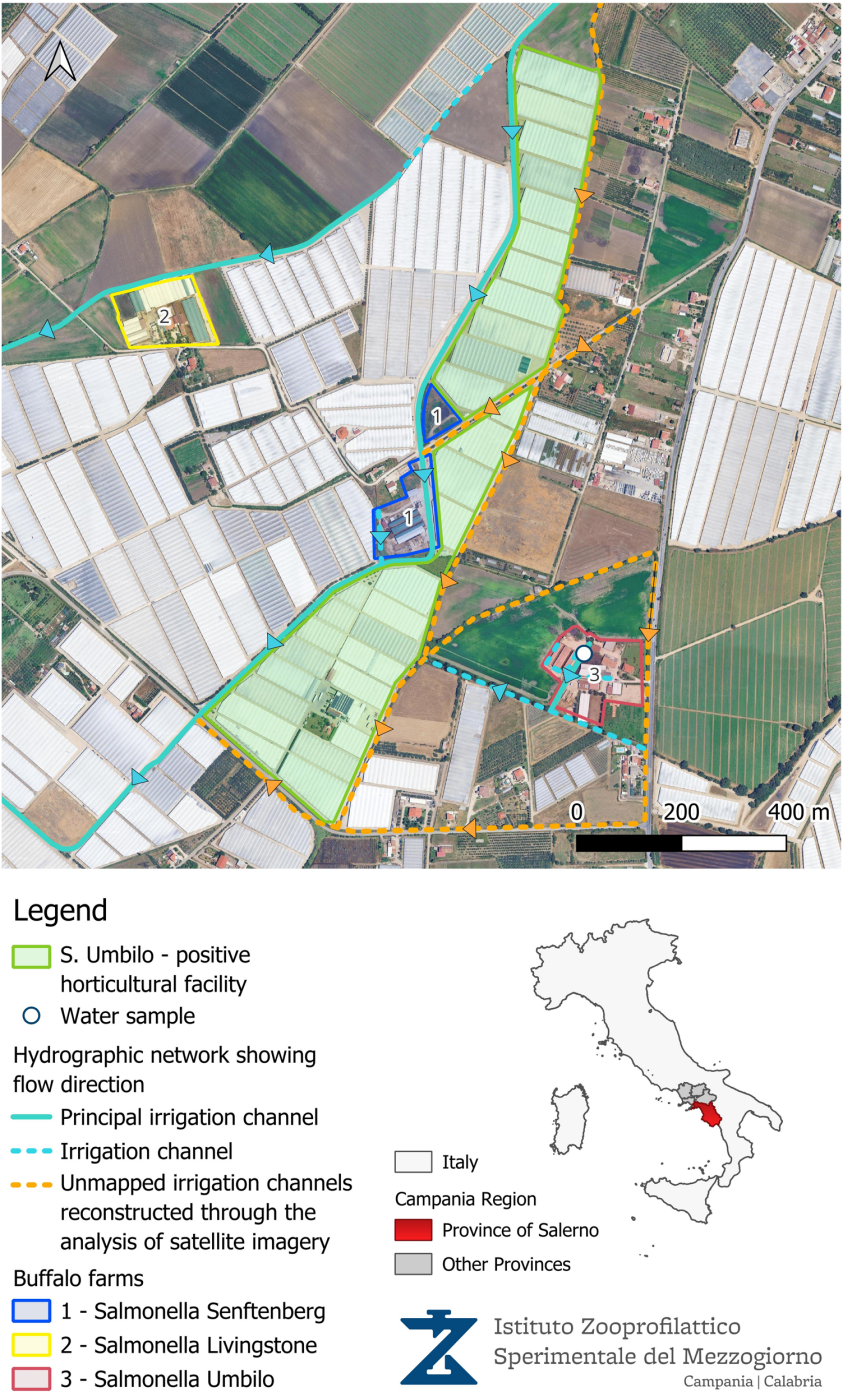
**Spatial distribution of the horticultural facility positive for *S*. Umbilo, irrigation channels, and buffalo farms positive for detected** ***Salmonella*** **serotypes.**

The serotyping of the isolated strains identified three distinct serotypes, with one serotype found in each of the three sampled farms: *S*. Senftenberg (*n* = 4), *S*. Livingstone (*n* = 10) and *S*. Umbilo (*n* = 11), respectively, as reported in Table 2 and Figure 1.

**Table 2:** ***Salmonella* spp. serotypes isolated from faecal samples of buffalo calves, Italy, 2024–2025 (*n*** **= 25 samples)**

*Salmonella* Serotypes	Farm 1	Farm 2	Farm 3	Total
*S.* Senftenberg	4	0	0	4
*S.* Livingstone	0	10	0	10
*S.* Umbilo	0	0	11	11
Total	4	10	11	25

Since *S*. Umbilo was recovered from buffalo calves’ faecal samples of one farm, close to the horticultural company producing the contaminated rocket salad and baby spinach linked to the outbreak described in RASFF Alert 2024.7033, sequencing was performed to assess the genomic correlation among these isolates.

**Figure 2:**
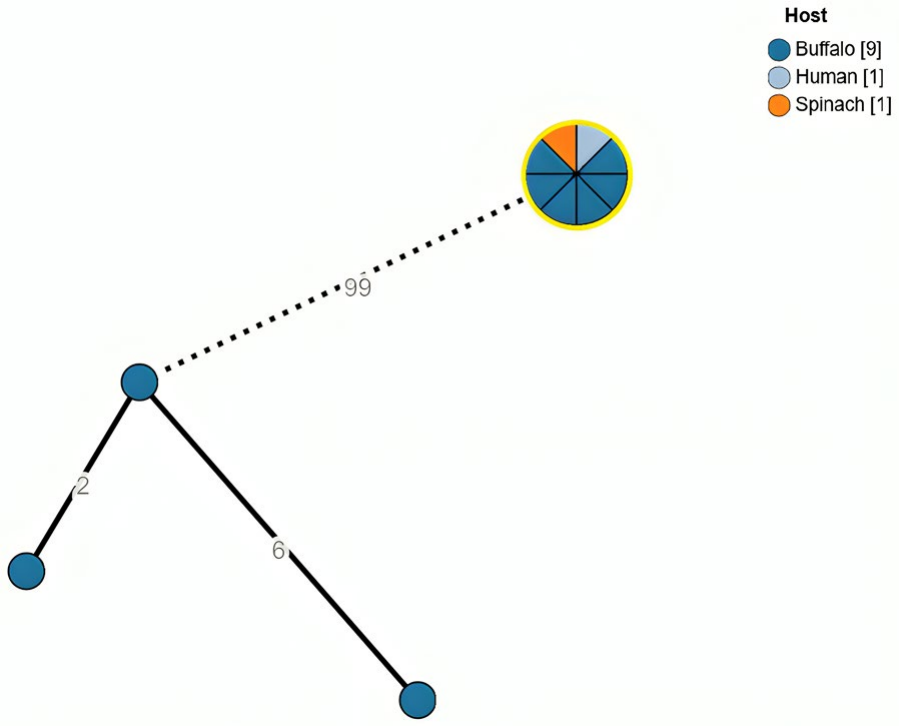
**Minimum spanning tree (MST) based on the cgMLST analysis of *Salmonella*** **Umbilo isolates.** The highlighted (yellow) circle composed of multiple samples (pie-chart node) represents isolates grouped according to the same cgMLST allelic profile, all at 0 allelic distance (0 AD) from each other. This cluster includes the clinical reference strain, the baby spinach strain and the isolates from the buffalo farm under investigation. Different colours indicate the various hosts from which the strains were isolated. Numbers on the branches represent the allelic distances (AD) between isolates. Dashed edges indicate branches with a length > 10 AD. Samples positioned at 99 AD were included only as a reference to illustrate allelic distances; they correspond to buffalo isolates collected between 2019 and 2020 in the same geographical region.

According to the results from the genomic comparisons based on cgMLST, the genetic sequences of six farm-isolated strains were fully comparable (0 AD) to those of the clinical case (ENA accession number ERR13934259) [1] and the baby spinach sample (NCBI BioSample ID SAMN44599062) [1], as shown in Figure 2.

A follow-up inspection at the affected farm revealed a connection between the farm and horticultural company through shared irrigation channels, which were highly susceptible to contamination from livestock faeces due to their proximity to housing areas. Water samples collected from these channels on two occasions, 15 days apart, later showed high levels of *E. coli* contamination (> 24.196 MPN/100 mL) but did not confirm the presence of *Salmonella* spp.

## Discussion

The investigations conducted within the three farms revealed the presence of three different *Salmonella* serotypes, all with zoonotic potential. However, our focus was primarily on *S*. Umbilo, which was repeatedly isolated from farm 3 in eleven faecal samples from buffalo calves that showed no clinical signs at the time of sampling.

Subsequent biomolecular characterisation confirmed that the isolated genotypes matched the one identified in individuals affected by the foodborne infection and in the contaminated rocket salad they consumed [1]. This finding suggests that the buffalo farm, located near the cultivated fields, may have played a role in the contamination of the rocket salad grown nearby (Figure 1).

To prevent further contamination, biosecurity and sanitation measures were implemented at all three farms. These included comprehensive cleaning and disinfection of animal housing facilities and sanitation of equipment using disinfectants effective against *Salmonella* spp. and following recommended conditions of use. In the calf barn, a sufficient number of pens was allocated to allow a 7-day sanitary break before introducing new-born calves, and colostrum was manually collected and administered to avoid the transmission of *Salmonella* spp. through direct contact with the mother. Rodent control and restrictions on access to housing areas by unauthorized personnel and other domestic or synanthropic animals were also implemented. Additional interventions were applied at the farm 3, including full coverage of ground-level, open irrigation channels near the buffalo housing areas and enhanced sanitation practices, with a particular focus on the calving area and the calf barn. These interventions led to a progressive reduction in *Salmonella* spp. positivity in buffalo calves, eventually achieving complete decontamination, as monitored through the follow-up conducted from January to April 2025. During the initial 2-month follow-up after the first positive detection at farm 3, three consecutive faecal samplings of 30 calves, performed at 7–8-day intervals, were systematically negative. This monitoring, carried out by the farm animal veterinarian, included both calves that had previously tested positive and those born during the observation period.

Furthermore, the use of an autogenous vaccine, formulated from the *S*. Umbilo strain previously isolated in the farm, has been planned to permanently eliminate the pathogen from the farm. The prompt implementation of these measures is expected to enhance the management of agricultural practices and mitigate the risk of any further contamination of vegetables cultivated in close proximity to the buffalo farms.

## Data Availability

Raw sequencing data, metadata, and genome assemblies for six Salmonella enterica serovar Umbilo isolates have been submitted to the European Nucleotide Archive (ENA) under study accession PRJEB89027. Sample accessions are ERS24299956—ERS24299961, with associated sequencing runs available under accessions ERR14904623, ERR14904624, ERR14904626—ERR14904629. Corresponding draft genome assemblies are available under analysis accessions ERZ26858594, ERZ26858628—ERZ26858632.
